# Investigating Correlations and the Validation of SMAP-Sentinel L2 and In Situ Soil Moisture in Thailand

**DOI:** 10.3390/s23218828

**Published:** 2023-10-30

**Authors:** Apiniti Jotisankasa, Kritanai Torsri, Soravis Supavetch, Kajornsak Sirirodwattanakool, Nuttasit Thonglert, Rati Sawangwattanaphaibun, Apiwat Faikrua, Pattarapoom Peangta, Jakrapop Akaranee

**Affiliations:** 1 Department of Civil Engineering, Faculty of Engineering, Kasetsart University, Bangkok 10900, Thailand; fengatj@ku.ac.th (A.J.); fengsvsu@ku.ac.th (S.S.); kajornsak.si@ku.th (K.S.); nuttasit.t@ku.th (N.T.); 2 Hydro-Informatics Innovation Division, Hydro-Informatics Institute, Ministry of Higher Education, Science, Research and Innovation, Bangkok 10900, Thailand; rati@hii.or.th (R.S.); apiwat@hii.or.th (A.F.); pattarapoom@hii.or.th (P.P.); jakrapop@hii.or.th (J.A.)

**Keywords:** soil moisture, remote sensing, SMAP, Sentinel-1, soil–water retention curve, validation, Thailand

## Abstract

Soil moisture plays a crucial role in various hydrological processes and energy partitioning of the global surface. The Soil Moisture Active Passive-Sentinel (SMAP-Sentinel) remote-sensing technology has demonstrated great potential for monitoring soil moisture with a maximum spatial resolution of 1 km. This capability can be applied to improve the weather forecast accuracy, enhance water management for agriculture, and managing climate-related disasters. Despite the techniques being increasingly used worldwide, their accuracy still requires field validation in specific regions like Thailand. In this paper, we report on the extensive in situ monitoring of soil moisture (from surface up to 1 m depth) at 10 stations across Thailand, spanning the years 2021 to 2023. The aim was to validate the SMAP surface-soil moisture (SSM) Level 2 product over a period of two years. Using a one-month averaging approach, the study revealed linear relationships between the two measurement types, with the coefficient of determination (R-squared) varying from 0.13 to 0.58. Notably, areas with more uniform land use and topography such as croplands tended to have a better coefficient of determination. We also conducted detailed soil core characterization, including soil–water retention curves, permeability, porosity, and other physical properties. The basic soil properties were used for estimating the correlation constants between SMAP and in situ soil moistures using multiple linear regression. The results produced R-squared values between 0.933 and 0.847. An upscaling approach to SMAP was proposed that showed promising results when a 3-month average of all measurements in cropland was used together. The finding also suggests that the SMAP-Sentinel remote-sensing technology exhibits significant potential for soil-moisture monitoring in certain applications. Further validation efforts and research, particularly in terms of root-zone depths and area-based assessments, especially in the agricultural sector, can greatly improve the technology’s effectiveness and usefulness in the region.

## 1. Introduction

Soil moisture is a critical factor in various fields of hydrology, agriculture, climate modeling, and land management. The temporal and spatial distribution of soil moisture in watersheds can affect the amount of water that enters streams, rivers, and groundwater, which, in turn, can impact the water availability for human consumption, agriculture, and other uses [1]. Wise management of soil moisture is thus critical for ensuring sustainable land use and for maximizing agricultural productivity. Soil-moisture data can be used to provide information about plant growth and health [2], nutrient availability, soil erodibility, as well as to optimize the irrigation schedule [3] and to aid in crop selection. Additionally, soil moisture is an important parameter in predicting climate-related disasters such as floods, landslides, and droughts. Early warning systems for such disasters often rely on accurate soil-moisture monitoring as part of their scheme.

As soil moisture significantly affects the partitioning of energy at the land surface, it exerts a considerable impact on the surface energy balance and atmospheric processes, such as surface temperature, surface evaporation, and transpiration. Knowledge of soil moisture can enhance the accuracy of weather forecasting and climate modeling at both global and regional scales. Remote-sensing techniques have been developed to measure soil moisture and related parameters [4], such as active and passive microwave remote sensing (e.g., Soil Moisture Active Passive, known as SMAP [5]), optical remote sensing for the Normalized Difference Vegetation Index (NDVI) [6], and thermal remote sensing (Moderate Resolution Imaging Spectroradiometer (MODIS) [7] or the Thermal Infrared Sensor (TIRS) [8].

SMAP is a NASA satellite mission launched in 2015, utilizing both active and passive microwave remote sensing to measure soil moisture with high spatial resolution [5]. Other satellites, such as Soil Moisture and Ocean Salinity (SMOS), also employ passive microwave techniques, particularly at the L-band frequency, enabling global mapping of near-surface (0–5 cm) soil moisture with 25 to 40 km spatial resolution and a temporal resolution of 2 to 3 days [4]. A study by Forgotson et al. [9] demonstrated several applications of SMAP soil-moisture products to improve soil-moisture-related monitoring and early warning systems in the United States (U.S.) through case studies. They reported that SMAP soil-moisture information could be utilized to enhance the capacity to predict snowmelt flooding by capturing antecedent soil-moisture conditions prior to freeze up and using this information to update snow water equivalent (SWE) estimates in the U.S. from 2015 to 2018.

In order to validate the SMAP soil-moisture product, Colliander et al. [10] presented the results from 34 candidate core validation sites in various parts around the world for the first eleven months of the SMAP mission. They indicated that the SMAP radiometer-based soil-moisture data product meets its expected performance of 0.04 m^3^/m^3^ volumetric soil moisture (unbiased root-mean-square error). Rahman et al. [11] also conducted a validation analysis of the SMAP L4 soil-moisture (SSM) data against in situ measurements obtained from various locations and land use types in the U.S. The authors suggested that SMAP L4 data can be a valuable tool for accurately mapping cropland inundation, providing useful information for flood monitoring and management. The study revealed that the SMAP data demonstrated the closest agreement with in situ measurements from the central Great Plains and cultivated croplands throughout the year. In a study conducted by Zhang et al. [12], the reliability of SMAP L-band radiometer data was also validated against in situ soil-moisture data from selected agriculture sites in the U.S. The previous study specifically focused on assessing the influence of spatial–temporal characteristics, particularly on land covers such as cultivated crops, deciduous/evergreen forest, and pasture/hay. Their work also explained a methodology to derive a soil wetness index from the time series of SMAP L-band brightness temperatures. The findings from their study indicated that the SMAP L4 products exhibited high reliability and demonstrated a significant impact of spatial–temporal characteristics, particularly on certain land cover types. In addition, the SMAP L4 soil-moisture data was found to be significantly affected by seasonal variation in these land covers. Zhou et al. [13], using the 43-station soil moisture over eastern China validated the satellite soil-moisture products from various sources, including Chinese FengYun 3C (FY3C), the level-2 neural network product of the European Soil Moisture and Ocean Salinity (SMOS), and the level-4 product of the U.S. Soil Moisture Active Passive (SMAP). They found the SMAP product to generally be the best among the three products in terms of the root-mean-square error (RMSE), unbiased RMSE, and correlation coefficient (R). Zhao et al. [14] compared NASA’s SMAP surface soil-moisture products to the High-Resolution Land Assimilation System (HRLDAS) surface-layer soil moisture for irrigation management purposes in Nebraska. They attempted to indirectly validate its root-zone soil-moisture (RZSM) product (~top 1 m) and suggested a simple calibration that was applied to the HRLDAS products by including the irrigation amount as one of its variables.

These previous studies clearly demonstrate the importance of regional and local validation of the SSM data prior to their application to any specific region worldwide. Such validation is essential to ascertain the reliability and suitability of the SSM data for use in particular geographical areas. The main challenge still arises from the significant disparity in measurement scales between satellite-based SSM (in kilometers) and in situ measurements (in centimeters). The penetration depth of the satellite measurement is normally less than 10 cm [15] while the in situ measurement can be made at any desired depth. This difference is influenced by factors such as land cover type, soil type, and the complex boundary conditions of the soil strata. This current study thus aims to validate the SSM products for Thailand by correlating the remote-sensing data with in situ soil-moisture measurements from 10 telemetry stations encompassing diverse soil types and land covers in Thailand. In addition, a detailed analysis of soil properties, including the particle size distribution, plasticity, organic content, thermal conductivity, porosity, and soil–water characteristic curves, was also conducted. These analyses were subsequently used in a multiple linear regression analysis to provide simple transfer functions for remote-sensing and in situ soil-moisture measurements. These transfer functions will serve as a baseline for more advanced models in the future.

In the paper, Section 2 provides a detailed description of the material and methods employed in this study. Subsequently, in Section 3, we present the analysis results derived from the in situ measurements and the validation of the SMAP soil moisture. Finally, Section 4 provides a comprehensive discussion of the results and summarizes the main findings obtained from this study.

## 2. Materials and Methods

### 2.1. Remote Sensing Soil Moisture

**Figure 1:**
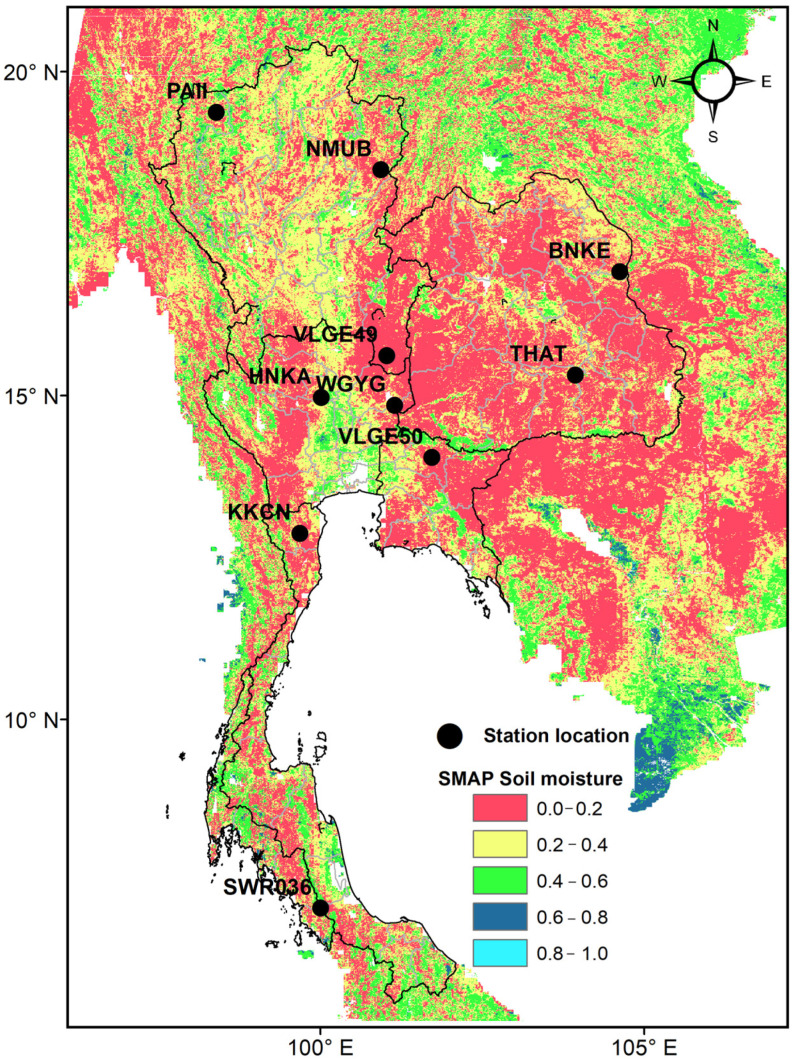
An example of a typical SMAP soil-moisture map (shaded color) and locations of the in situ soil-moisture stations (black circles) used in the study.

This study utilizes the SMAP radiometer/Copernicus Sentinel-1 soil-moisture product (L2_SM_SP) of the National Aeronautics and Space Administration (NASA) (Washington, DC, USA), designated as SPL2SMAP_S SMAP/Sentinel-1 L2 Radiometer/Radar 30-Second Scene 3 km EASE-Grid Soil Moisture, Version 3 [16,17]. Being a Level-2 (L2) soil-moisture product, it provides estimates of land surface conditions retrieved by both the SMAP radiometer during the 6:00 a.m. descending and 6:00 p.m. ascending half-orbit passes and the Sentinel-1A and -1B radar. The soil-moisture data was derived using SMAP L-band brightness temperatures and Copernicus Sentinel-1C-band backscatter coefficients. Subsequently, the datasets were resampled to an Earth-fixed, cylindrical 3 km Equal-Area Scalable Earth Grid, Version 2.0 (EASE-Grid 2.0) [16,17]. For Thailand, the available SMAP L-band data mostly corresponds to nighttime, while the Sentinel-1 data covers both daytime and nighttime observations. In this study, the soil-moisture data from 149 files spanning approximately one month are stitched together to cover all the pixels across Thailand. An example of the monthly soil-moisture map that can be produced is shown in Figure 1.

### 2.2. In Situ Soil-Moisture Monitoring

In situ soil-moisture data used to validate the SMAP soil moisture were collected from 10 telemetry monitoring stations installed throughout various locations in Thailand. These stations are extensions of the existing telemetry weather stations belonging to the Hydro-Informatics Institute (HII) of Thailand. The locations of these stations were carefully selected to represent a variety of soil types, geographical features, and climate conditions in Thailand, offering valuable data for understanding soil-moisture behavior in more topographically varied landscapes.

The distribution of the monitoring stations across diverse topographic and land-use settings in Thailand is presented in Figure 1 and summarized in Table 1. As seen, five stations (HNKA, WGYG, VLGE50, BNKE, and THAT) were situated on flat plains, while three stations (KKCN, PAII, and SWR036) were located in hilly terrains, and the other two stations (VLGE49 and NMUB) were on undulating plains.

From Table 1, the telemetry stations exhibited a wide variation in annual rainfall, ranging from the lowest recorded value of 983.0 mm (KKCN station) to the highest recorded value of 2386.0 mm (SWR036 station). This rainfall variability reflects the diverse hydrological conditions across the monitored regions and can significantly influence soil-moisture patterns. Moreover, the land-use types surrounding these stations encompassed various categories, including agriculture (field crops or rice paddy), perennial and urban areas, and forested regions. Understanding soil-moisture features in these distinct land-use types is also essential for assessing the impacts of different land covers on soil-moisture dynamics.

**Figure 2:**
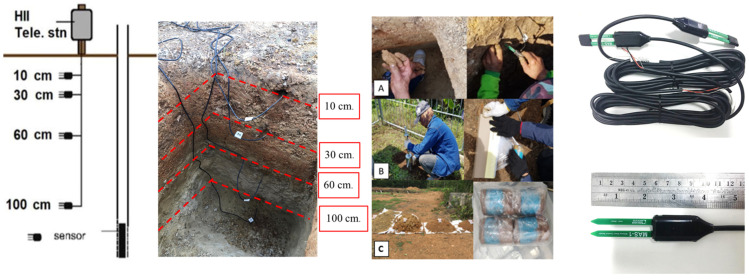
Test pit and installation of soil-moisture sensors: (**A**) shows installation of a SM sensor along the side wall of the pit, while (**B**,**C**) show collecting soil samples at a site.

To delineate the soil stratigraphy within the uppermost 1 m layer at the monitoring station, a test pit was dug to collect in situ soil samples (both disturbed and undisturbed samples) for further testing. In situ field classification was performed based on soil color and texture, enabling the description of soil stratigraphy, which was divided into 2–3 layers over the 1 m depth. The soil-moisture sensors (MAS-1, METER Group, Pullman, WA, USA with a standard 2-wire, 4 to 20 mA type) were installed at depths of 0.10, 0.30, 0.60 and 1.00 m along the side wall of the pit, as shown in Figure 2. During the sensor installation process, each sensor was horizontally inserted into a pre-bored hole created on the test pit side-wall using a dummy sensor of the same size to minimize any potential damage to the actual sensor. Great care was taken to minimize soil disturbance during installation. However, when the soil was dry and compacted, a small hole (1–2 cm in diameter and about 10 cm long) was created horizontally in the test pit wall. The excavated soil was then moistened and carefully backfilled into the hole before inserting the sensor. Following the installation of all the sensors on the pit wall, the pit was backfilled with excavated soil to restore it to its original condition.

The MAS-1 sensor employs capacitance/frequency domain technology to determine local volumetric water content (θ) by measuring the soil’s dielectric constant. In this study, the volumetric water content obtained from the in situ sensor is referred to as the local water content (θ), while the water content derived from remote sensing is called SMAP surface soil-moisture content (SSM). For site-specific calibration of the soil-moisture sensors, soil samples were collected from each delineated soil layer. Undisturbed soil samples were collected using a soil core sampler (63 mm inner diameter) from each soil layer to determine porosity, the soil–water retention curve, hydraulic conductivity, and thermal conductivity. Disturbed soil samples were used for basic classification tests, such as wet sieve analysis, the hydrometer test, and the determination of organic content, specific gravity, and Atterberg’s limits.

### 2.3. Laboratory Testing of Soil

**Figure 3:**
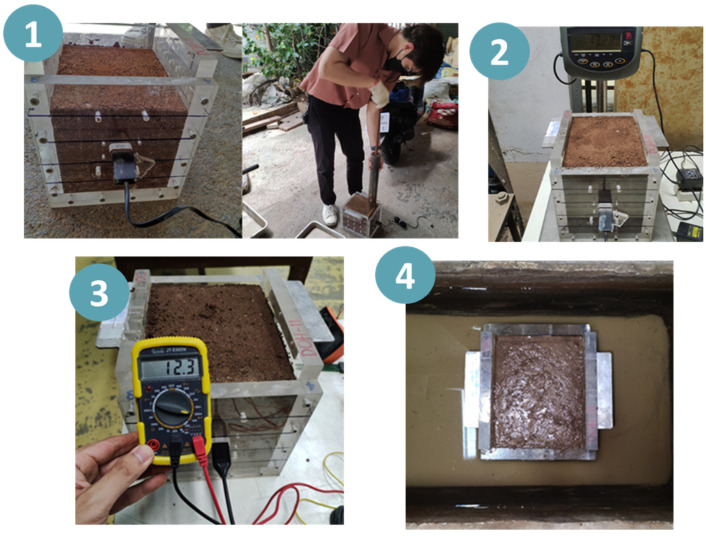
Calibration process for soil-moisture sensors: (**1**) sample preparation, (**2**) sample weighing, (**3**) sensor reading at different compaction moisture contents, and (**4**) final soaking.

Figure 3 shows the calibration box (with inner dimensions of 16.2 × 18.2 × 20.1 cm^3^) and the procedure used to establish the relationship between the sensor output (mA) and volumetric water content (θ $=V_{w}/V$, where $V_{w}$ is the volume of water and V is the total volume of soil). This specific size of the calibration box was chosen to minimize boundary effects on the moisture sensor signal, as suggested by Suwansawat et al. [18]. To replicate field conditions as closely as possible, the soil sample was re-compacted in the calibration box to achieve the same dry unit weight as that of an undisturbed sample. A number of soil samples were compacted to different moisture contents and the sensor output was measured accordingly for each moisture level. The final stage of calibration involved soaking the samples for several days until the sensor reading stabilized, indicating a saturated condition.

Three undisturbed cylindrical samples (63 mm in diameter and about 25 mm in height) were obtained from each soil layer and utilized for conducting the soil–water retention curve, hydraulic conductivity, and thermal conductivity tests. The soil–water retention curve (SWRC) procedure involved measuring the soil suction at different water contents in a drying path, as explained in Barus et al. [19] and Shrestha et al. [20]. The test began by soaking the soil sample for several days to reach full saturation and then gradually drying the sample. At each drying stage, the soil suction, weight, and dimension of the soil sample were measured under equilibrium conditions. For suction measurements, three methods were employed; namely, the miniature tensiometer for matric suctions less than 100 kPa, the pressure plate for matric suctions between 200 to 1500 kPa, and the isopiestic technique (salt solution equilibrium) for total suctions greater than 1500 kPa. Additionally, the porosity and volume changes in the soil sample were also monitored during the SWRC test. The hydraulic conductivity and thermal conductivity tests were carried out on the undisturbed samples in a saturated condition following the American Society for Testing and Materials (ASTM) standards as described in [21] and [22], respectively. Other index properties, including specific gravity, Atterberg’s limits, and from the wet sieve analysis and hydrometer test were determined based on the ASTM standards [23,24,25]. The organic content was determined using the chronic acid titration method [26].

### 2.4. Basic Soil Properties

Table 2 summarizes the basic soil properties and soil classification (based on the unified soil classification system, USCS) of all the prescribed soil layers in all the telemetry stations. At four stations (KKCN, VLGE49, VLGE50, PAII), the soils were described as clays (CL and CH) of varying plasticity indices (PI) ranging between 5.7 to 37.9. Silty soils (classified as ML and MH) were found at three stations; namely, WGYG, NMUB, and PAII. Silty sand and clayey sand were present at the remaining four stations, i.e., HNKA, BNKE, THAT, and SWR036. In most cases, the organic content of the soils was lower than 20 g/kg, which was considered low, with the exception of WGYG (0–50 cm) and PAII (0–10 cm), which had higher organic matter varying from between 30 to 35 g/kg.

The porosity of HNKA and THAT soils were relatively lower than that of the others (<0.35), which indicated the compaction of the soil resulting from human activities, e.g., preparation of the land for urban uses or any other development. It should also be noted that at the HNKA station, the soil collected at the telemetry station was classified as clayey sand and silty sand, while the soil map of Land Development Department (LDD) of Thailand indicated that the soil in the area should be clay (Unit 4, Cn). This discrepancy was due to the fact that the soil-moisture sensors were required to be installed near the telemetry station, which was located on an engineering sandy soil fill and may not be representative of the clay that extended in most of nearby rice paddy. Such a discrepancy between the properties of the collected soil and the information from the LDD soil map was to be expected due to spatial variability of the in situ soil.

### 2.5. Soil-Moisture Sensor Calibration

**Figure 4:**
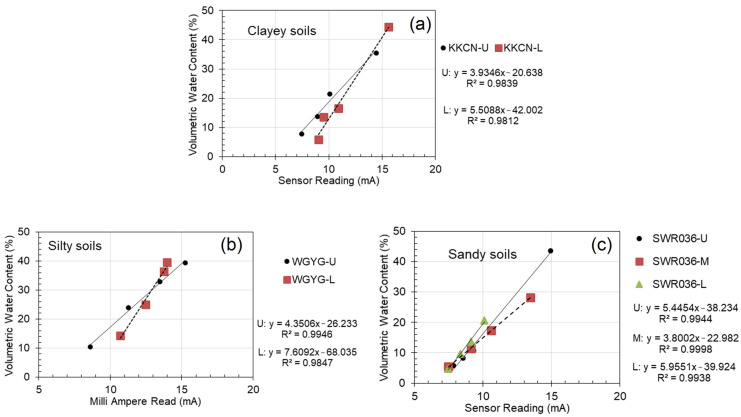
Examples of sensor calibration equations for different soil types: (**a**) clayey soils, (**b**) silty soils, and (**c**) sandy soils.

The soil-moisture sensor required a soil-specific calibration so that a reliable reading could be achieved. Figure 4 shows some examples of sensor calibration results for different soil types. Table 3 summarizes the coefficients of the calibration equation for a soil-moisture sensor that takes a linear form as follows:

(1) θ=ax+b

where x is the sensor reading in milliamps (mA), a and b are the fitting parameters from the linear regression, and θ is the volumetric water content (%). The coefficient of determination, R^2^, also summarized in Table 3, was found to range between 0.980 to 0.999, which indicated a satisfactory correlation and accuracy. It is noteworthy that the default calibration equation provided by the manufacturer of the MAS-1 sensor can give very different values of soil moisture compared with the soil-specific equation (Table 2), varying by about 7% (in terms of θ) on average, and the maximum discrepancy in θ can be as much as 22.9%. It is thus of utmost importance to determine the soil-specific calibration equation in the laboratory using the in situ soil for a reliable measurement.

In this study, multiple linear regression analyses were conducted to model the relationships between the fitting parameters (a and b, taken as dependent variables) and soil properties (taken as independent variables), as shown in Equations (2) and (3).

(2) $$a=\alpha_{c}+\alpha_{1}X_{1}+\alpha_{2}X_{2}+\alpha_{3}X_{3}+\alpha_{4}X_{4}+\alpha_{5}X_{5}+\alpha_{6}X_{6}+\alpha_{7}X_{7}$$

(3) $$b=\beta_{c}+\beta_{1}X_{1}+\beta_{2}X_{2}+\beta_{3}X_{3}+\beta_{4}X_{4}+\beta_{5}X_{5}+\beta_{6}X_{6}+\beta_{7}X_{7}$$

where $X_{1}$ is the plasticity index (for non-plastic soil, $X_{1}$ is taken as zero), $X_{2}$ is the % gravel, $X_{3}$ is the % sand, $X_{4}$ is the % silt, $X_{5}$ is the % clay, $X_{6}$ is the porosity (unitless), and $X_{7}$ is the organic content (g/kg), as summarized in Table 2. The fitting parameters $\alpha_{i}$ and $\beta_{i}$ (i = 1, 2, …, 7) are obtained from multiple linear regression analyses as summarized in Table 4. These relationships are useful in the case where there is no site-specific sensor calibration test and only information on the soil texture, porosity, and organic content is available. Then the sensor calibration coefficients (*a* and *b*) can be predicted using the known basic soil properties $\left(X_{1},\ldots ,X_{7}\right)$. The coefficient of determination (R^2^) from the multiple linear regression analysis of the *a* and *b* parameters was 0.748 and 0.721, respectively.

### 2.6. Hydraulic and Thermal Properties

**Figure 5:**
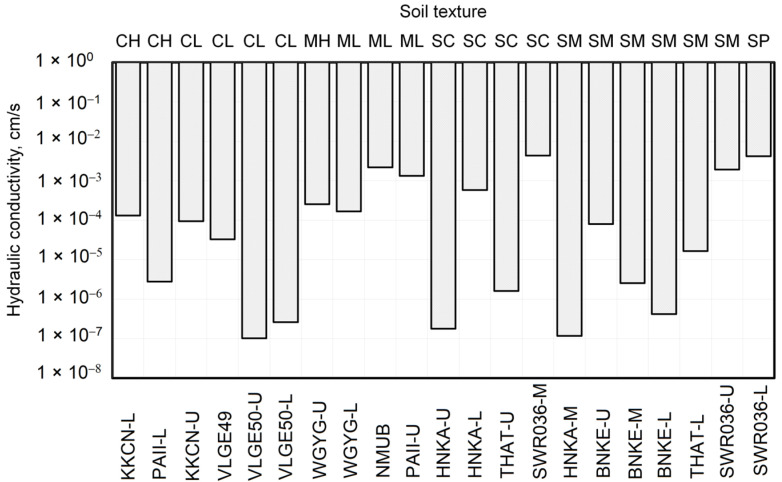
Hydraulic conductivity of the undisturbed core sample in a station and its soil texture characteristics.

Figure 5 illustrates the hydraulic conductivity values of the undisturbed core samples (from Table 3) categorized by soil textures, ranging from fine-grained (CH) to coarse-grained (SP). The initials are shown according to the Unified Soil Classification System (CH = high-plasticity clay; CL = low-plasticity clay; MH = high-plasticity silt; ML = low-plasticity silt; SC = clayey sand; SM = silty sand; and SP = poorly graded sand). The variation of hydraulic conductivity for the clay soils (CH and CL) ranged between 10^−7^ to 10^−4^ cm/s (about three orders of magnitude), while that of silts (ML and MH) were between 10^−4^ to 10^−3^ cm/s (one order of magnitude). The upper range of the hydraulic conductivity of clay was relatively high (10^−4^ cm/s) due to the aggregated structure of the material. The clayey and silty sands (SC and SM) had hydraulic conductivities that varied to a greater extent, between 10^−7^ to 10^−3^ cm/s, which reflected the influence of the clay particles present. The poorly graded sand (SP) had the highest value of hydraulic conductivity of 4.2 × 10^−3^ cm/s. The observed variations in hydraulic conductivities clearly indicate that soil textures alone are insufficient to fully explain the range of hydraulic conductivities.

**Figure 6:**
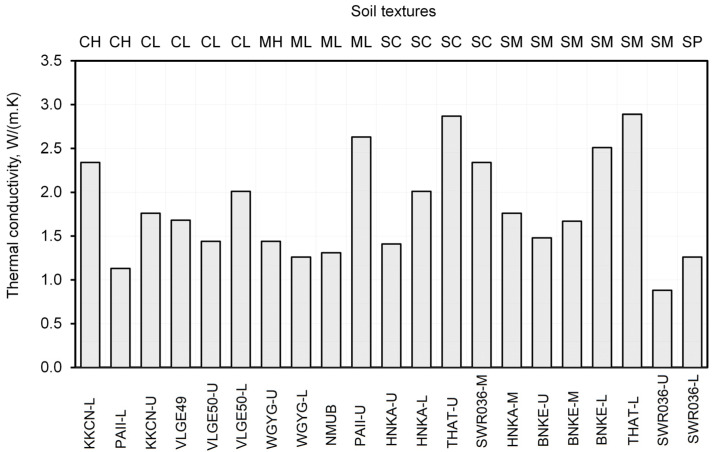
Thermal conductivity of the undisturbed core sample in the saturated condition of a station and its soil texture characteristics.

Figure 6 shows the thermal conductivity (λ) values (from Table 3) of the undisturbed core samples by soil textures. The values of λ ranged from 0.88 to 2.89 W/(m·K), which is within the typical range observed for soils. Various factors, such as soil mineral, porosity, moisture content, and organic content, can directly influence the thermal conductivity of the soil [27]. Notably, soils with higher quartz content are normally of greater thermal conductivity. Three soils, namely PAII-U, THAT-U, and THAT-L, exhibit thermal conductivities greater than 2.5 W/(m·K) and can be classified as silt (ML), clayey sand (SC), and silty sand (SM), respectively. It is likely that these soils have a higher proportion of quartz in their composition. However, it is important to note that there has been no mineral composition test conducted to verify this assumption.

### 2.7. Soil–Water Retention Curves

**Figure 7:**
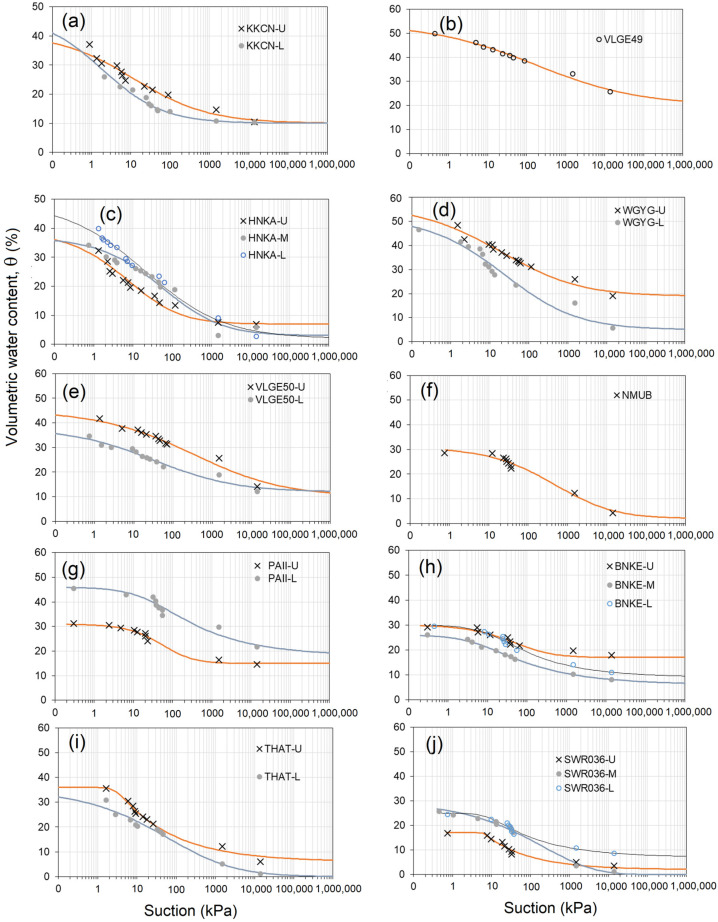
Soil–water retention curves estimated for the stations: (**a**) KKCN, (**b**) VLGE49, (**c**) HNKA, (**d**) WGYG, (**e**) VLGE50, (**f**) NMUB, (**g**) PAII, (**h**) BNKE, (**i**) THAT, and (**j**) SWR036.

Soil–water retention curves (SWRCs) of all the soils in this study are shown in Figure 7. It is noted that the curves represent the drying paths followed by the soil samples. These SWRCs were plotted as the volumetric water content, θ (%), against suction, s (kPa), together with curve fitting using the Van Genuchten [28] model, as shown in Equation (4).

(4) $$\theta =\left(\theta_{s}-\theta_{r}\right){\left[\frac{1}{1+{\left(p\cdot s\right)}^{n}}\right]}^{m}+\theta_{r}$$

where $\theta_{s}$ is the saturated volumetric water content, $\theta_{r}$ is the residual volumetric water content, p is the fitting parameter that is inversely proportional to the air-entry suction of the soil, and m and n are the fitting parameters related to the pore-size distribution. Table 5 summarizes the Van Genuchten parameters obtained from best fitting of the experimental data points for all the soils tested in this study. The wide range of SWRC fitting parameters in this study is attributed to the diverse range of soils encountered. These parameters provide indications about various soil properties such as the pore-size distribution, soil texture, soil aggregation structure, and organic content. It is important to note that these properties can vary spatially with depth and temporally, depending on the land-use type.

It is worth mentioning that hysteresis can significantly impact the SWRC. However, the parameters presented here solely pertain to the drying path, which involves increasing suction or decreasing water content. The wetting SWRC was not within the scope of this study and, therefore, not considered in the analysis. The drying SWRC is particularly relevant for analyzing drought events rather than flooding. By focusing on the drying path, these parameters can provide valuable insights into the water availability and soil-moisture retention during dry periods. The information on SWRC presented here provides the fundamental link between soil-moisture content and suction, which is a direct measure of the energy state of water in the soil. It reflects how tightly the water is held in the soil and how difficult it is for plants to extract it. The SWRC thus has the potential to expand the use of satellite soil moisture to a wider range of applications.

## 3. Results

### 3.1. Correlations and Calibration of SMAP-Sentinel and In Situ Soil Moisture

#### 3.1.1. Effect of the Temporal Variation in Soil Moisture

**Figure 8:**
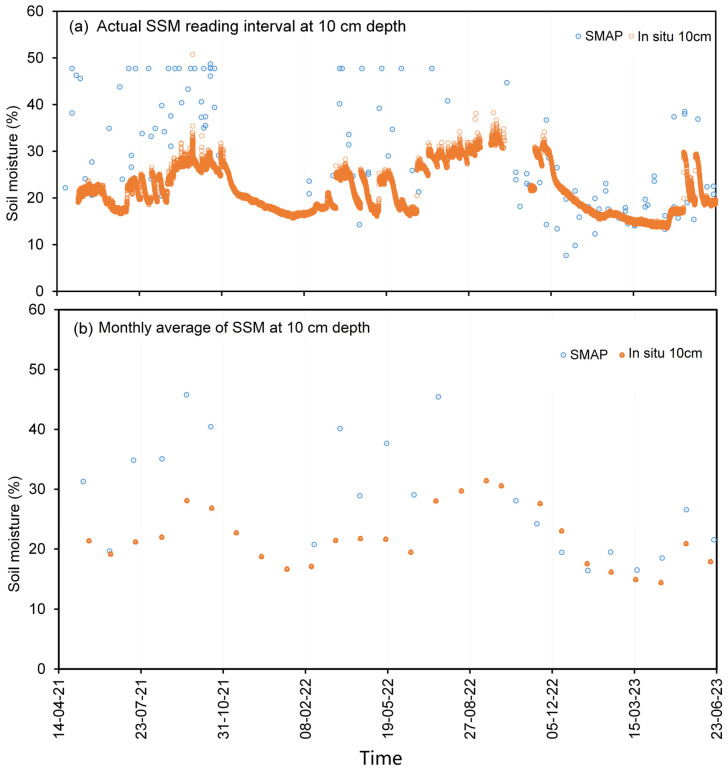
Variations in SMAP and in situ soil moisture (10 cm depth) data from Station VLGE49 from (**a**) the actual measurement reading intervals and (**b**) the monthly average values of the SSM.

**Figure 9:**
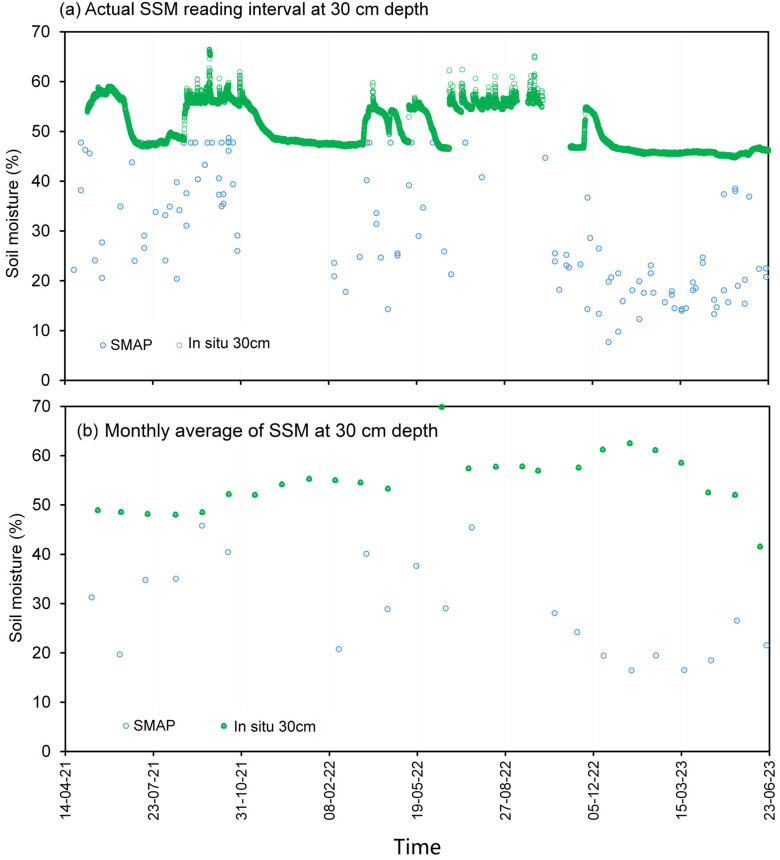
Variations in SMAP and in situ soil moisture (30 cm depth) from Station VLGE49 from (**a**) the actual measurement reading intervals and (**b**) the monthly average values of the SSM.

The temporal resolution disparity between the SMAP product, which is available in intervals ranging from 3 to 8 days, and the high-frequency hourly measurements of in situ moisture content necessitates the adoption of an averaging approach to establish correlations between these datasets. To elucidate this, Figure 8a and Figure 9a vividly depict the original soil-moisture variations at depths of 10 cm and 30 cm, respectively, for Station VLGE49. It is intriguing to observe that the SMAP soil moisture (SSM) exhibits significantly larger and more oscillatory amplitudes in comparison with in situ readings. Particularly noteworthy is the observation that, at a depth of 30 cm (as depicted in Figure 9), in situ moisture values surpass those of the SSM, suggesting the potential influence of diverse factors such as soil depth, interactions with groundwater, and spatial heterogeneity on the measurement outcomes.

**Figure 10:**
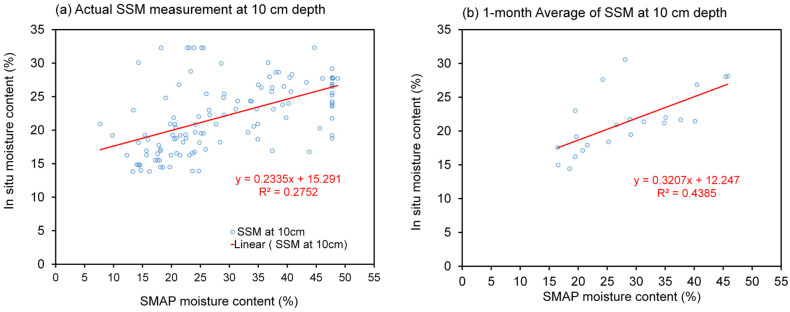
Correlation between of SMAP and in situ soil moisture (10 cm depth) for Station VLGE49 using (**a**) actual soil moisture measurement reading times and (**b**) the monthly average of the SSM.

**Figure 11:**
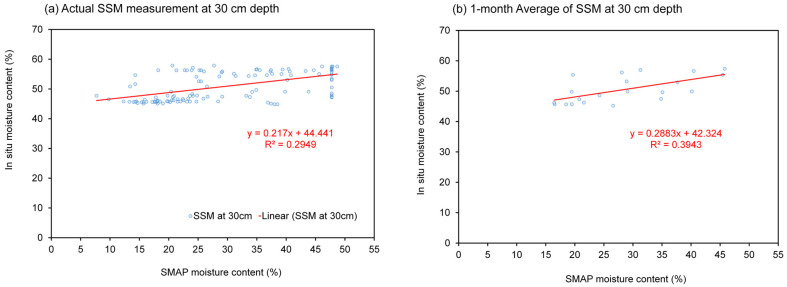
Correlation between SMAP and in situ soil moisture (30 cm depth) for Station VLGE49 using (**a**) actual soil moisture measurement reading times and (**b**) the monthly average of the SSM.

Introducing a 1-month averaging methodology, as illustrated in Figure 8b and Figure 9b, imparts a smoothing effect on the temporal profile of the soil moisture, thereby attenuating the magnitude of fluctuations. This smoothing effect culminates in an advantageous enhancement of the correlation between the SMAP product and the in situ measurements, as discernible in Figure 10 and Figure 11, which depict the relationship between the soil moisture at depths of 10 cm and 30 cm, respectively. It is acknowledged that the increase in the R-squared value as a result of monthly averaging was moderate (e.g., from 0.2752 to 0.4385 for a 10 cm depth). However, it is important to highlight that this improvement, although modest, is indicative of the positive impact of the smoothing process on the obtained results. In subsequent analyses, the utilization of one-month average soil moisture predominantly guided the exploration of correlations between the SSM and in situ measurements.

#### 3.1.2. Linear Correlation Coefficients and Soil Properties

The correlation between the SSM and in situ soil moisture based on 1-month average values can be expressed using the following linear equation:

(5) θ=M · SSM+C

This correlation was determined for all ten telemetry stations, and the fitting parameters (M,C) are summarized in Table 6 and Table 7, together with the coefficient of determination (R^2^). For three stations (KKCN, PAII and SWR036), the correlations were unreliable, due both to the excessive fluctuation in the SMAP data and malfunctions in the in situ moisture sensors. The correlation coefficients are not presented for these stations. Among the other seven stations, the coefficients of determination range between 0.13 to 0.58, which show a varying degree of fitting. The relatively poor fitting between the SMAP soil moisture and the in situ measurement is expected to be related to the disproportionate measurement areas, which will be discussed in the next section. The slope (M) of the linear equation is mostly below unity, which suggests that the SSM varies with a larger amplitude than does the in situ soil moisture. The intercept (C) is also greater than zero for all cases, indicating that even when the SMAP surface moisture tends to zero, some residual water content still remains in the surface soil. This kind of correlation is valuable for site-specific purposes and can be extended to sites with similar conditions.

The values of M and C are further correlated to a soil’s physical properties ($X_{1},\ldots ,X_{7}$), as explained in Section 3.1. The multiple linear regressions for M and C can be expressed in Equations (6) and (7), respectively:

(6) $$M=D+a_{1}X_{1}+a_{2}X_{2}+a_{3}X_{3}+a_{4}X_{4}+a_{5}X_{5}+a_{6}X_{6}+a_{7}X_{7}$$

(7) $$C=E+b_{1}X_{1}+b_{2}X_{2}+b_{3}X_{3}+b_{4}X_{4}+b_{5}X_{5}+b_{6}X_{6}+b_{7}X_{7}$$

where D, $a_{i}$, and $b_{i}$ (i = 1, 2, 3, …, 7) are curve-fitting parameters obtained from the regression analysis as summarized in Table 6. The coefficient of determination (R^2^) from the multiple linear regression analysis of the M parameter and the C parameter was 0.933 and 0.847, respectively. Provided that the basic soil properties ($X_{1},\ldots ,X_{7}$) are known at the site, the in situ soil moisture can then be predicted using the SMAP soil moisture and Equations (5)–(7). The estimated parameters given by the regression are summarized in Table 8.

#### 3.1.3. Upscaling of SMAP, In Situ Soil Moisture and Validation for Croplands

As discussed earlier, the relatively poor correlation between the SMAP soil moisture and in situ soil moisture at any specific telemetry station was largely attributed to the disproportionate measurement areas (1 sq·km for the SMAP soil moisture versus 0.01 sq·m for the in situ soil moisture) as well as to the spatial and temporal variations in in situ soil moisture. Land-cover types have also been shown to play a significant role in the correlation between the two kinds of measurements. Croplands generally demonstrated a better correlation due to the greater uniformity of the land cover and thus in situ soil moisture within the SMAP measurement pixels compared with other land covers. In this study, the MODIS land-cover-type product obtained from the International Geosphere–Biosphere Program (IGBP) was used to classify the land cover at the telemetry station in each SMAP measurement pixel. Seven stations are located in the croplands, namely PAII, VLGE49, BNKE, THAT, VLGE50, WGYS, and HNKA, while Station NMUB is in the woody savannas, SWR036 is in the evergreen broadleaf forests, and KKCN falls within the grassland type. Only data from seven stations in the croplands were used in further analyses. The average values of the soil moisture from all seven stations in the croplands were used in order to upscale both the SMAP and in situ soil moisture data, thus generalizing the soil moisture to represent the climate regimes of the cropland regions in Thailand.

**Figure 12:**
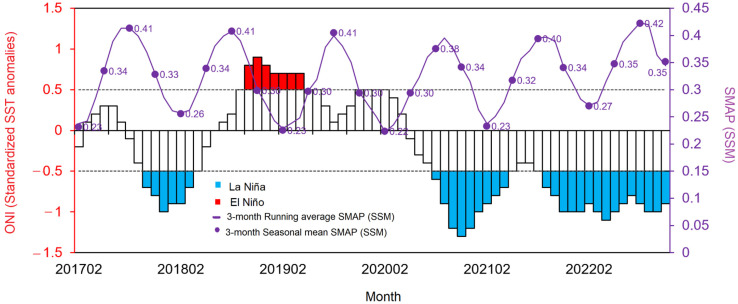
Average SMAP surface soil moisture from cropland areas and the ONI index. Dash lines represent values of ONI that commonly used as a threshold for El Niño (>+0.5°C) and La Niña (<−0.5°C).

In Figure 12, the variations in the average SMAP moisture values obtained from the seven measurement pixels in croplands (representing all seven telemetry stations) are depicted for the period between 2017 and 2022. These SMAP moisture values were calculated using the 3-month running average approach. This method aligns with the averaging period of the Oceanic Niño Index (ONI), which is also displayed in the figure. The Oceanic Niño Index (ONI) is NOAA’s primary index for tracking the ocean part of ENSO, the El Niño–Southern Oscillation climate pattern that indicates the difference from average in the surface waters of the east–central tropical Pacific. Also shown in the figure are the periods of El Niño (ONI ≥ 0.5) and La Niña (ONI ≤ 0.5). The average SMAP moisture calculated using the seasonal mean approach is also shown for comparison. The episodes of La Niña in 2018, 2021, and 2022 (shown as blue bar charts) contributed to the increase in soil moisture in these years, while the El Niño in 2019 (red bar charts) gave rise to a decrease in the soil moisture. The rainy season SMAP moisture reached the maximum value of 0.42 in 2022 (La Niña), while the minimum dry season SMAP moisture was 0.22 in 2020 (one year after El Niño).

Given our understanding of the influence of the El Niño–Southern Oscillation (ENSO) on the regional climate, it is reasonable to suggest that variations in the 3-month ENSO index may affect the surface soil moisture in Thailand over a corresponding 3-month period, primarily through its impact on rainfall variation. Torsri et al.’s recent study [29] highlights a significant negative response due to the ENSO in most of Thailand’s regions. Thus, the alignment between changes in the 3-month ENSO average and the SSM given by the present study implies that ENSO variability likely plays a role in modulating soil-moisture content in Thailand by means of its influence on ENSO-induced rainfall fluctuations.

**Figure 13:**
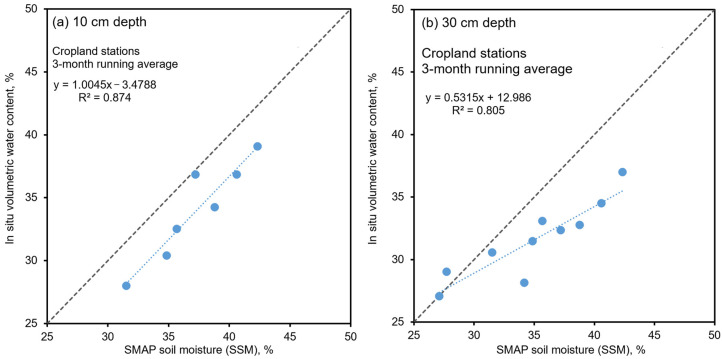
Relationships between SMAP soil moisture (SSM) and in situ volumetric water content, using data averaged between 7 stations in croplands, and averaged for each three-month running period spanning 2021 to 2022.

Based on the findings that the three-month running average period resulted in a reasonable agreement between the soil moisture and the Oceanic Niño Index (ONI), this averaging approach was adopted to determine the correlation between the in situ and the SMAP soil-moisture (SSM) data. The study utilized in situ soil-moisture data collected from seven stations situated in croplands. The data was averaged both spatially, between seven stations, and temporally, considering the three-month running period spanning 2021 to 2022. Figure 13 shows the relationship between the average SSM and the in situ water content, θ, at depths of 10 and 30 cm. The data was fitted using a linear model, yielding R-squared values ranging from 0.83 to 0.87, indicating a strong correlation between the SSM and the in situ *θ* when averaged using such approach. This is an important first step required for the further development of upscaling and downscaling models for soil-moisture measurements in Thailand. At a depth of 10 cm, the correlation revealed a systematic transformation shift of approximately −3%, suggesting that the SSM tended to overpredict in situ moisture (*θ*). However, the slope of the linear equation remained close to 1, indicating a relatively consistent relationship. Similarly, at the 30 cm depth, the correlation indicated SSM’s tendency to overpredict the in situ *θ*, with the slope of the linear equation measuring approximately 0.53.

One of the objectives of this research project was to produce a monthly surface soil-moisture data to be used for climate modeling and for improving weather forecasting at a national scale. The linear models presented earlier in Figure 13, using the three-month running average and cropland data, were then utilized to adjust the original monthly SMAP data to be more accurate and suitable for Thailand’s specific conditions. However, it is essential to acknowledge that extending the model calibrated with cropland data to all land-cover types in the country might introduce some inherent errors.

**Figure 14:**
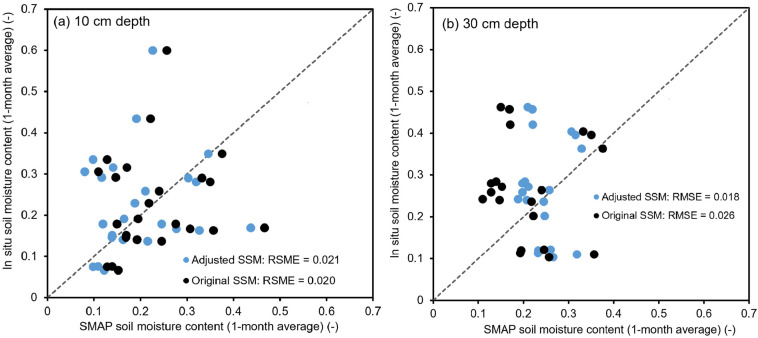
Relationship between the original and the adjusted 1-month average SMAP soil moisture (SSM) and in situ volumetric water content for all 10 stations (**a**) at the 10 cm depth and (**b**) at the 30 cm depth.

To assess the effectiveness of the SSM adjustment, we compared the monthly SMAP soil moisture values with and without model adjustment against the one-month average in situ soil moisture (θ_month_) from the ten telemetry stations between January and May 2023, as shown in Figure 14. We calculated the root-mean-square errors (RMSEs) to demonstrate the correlation improvements that could be expected from the SMAP SSM adjustment. For the case of the 10 cm deep in situ soil moisture, the linear model adjustment of the SMAP SSM did not significantly improve the accuracy of the correlation. The RMSE values were comparable, measuring 0.021 and 0.020 for the adjusted and original SMAP SSMs, respectively (Figure 14a).

However, a promising trend was observed for the adjusted SMAP SSM at the depth of 30 cm (Figure 14b). The RMSE value decreased from 0.026 for the original SMAP SSM to 0.018 after adjustment. This suggests that the adjustment technique holds potential for enhancing the accuracy at greater soil depths. Overall, while the linear model adjustment of the SMAP SSM showed mixed results, it has demonstrated the ability to improve the correlation in certain scenarios, particularly at greater soil depths. Further investigation and refinement of the models might be necessary to enhance the accuracy of the surface soil-moisture data across various land-cover types in Thailand.

## 4. Discussion and Conclusions

The present study undertakes an in-depth exploration of the intricate interplay between remotely sensed SMAP data and in situ soil-moisture measurements, with the overarching goal of advancing our comprehension of soil-moisture dynamics. This multifaceted investigation encompasses an array of aspects, including temporal variations, correlation coefficients, and the impact of soil properties. Collectively, these facets contribute to a comprehensive understanding of the complex process of soil-moisture estimation.

One of the central considerations in this study revolves around addressing the discrepancies between the SMAP data and the in situ measurements spanning the years 2021 to 2023. This discrepancy stems from the inherent differences in temporal resolutions between the two datasets. The solution lies in employing a monthly averaging approach, which serves to attenuate the inherent fluctuations in soil moisture and thus enhance the reliability of the correlation analysis. The observed disparities in soil-moisture variations underscore the multifaceted nature of soil-moisture dynamics. Interestingly, SMAP soil moisture exhibits more pronounced amplitude variations compared with in situ readings. A noteworthy phenomenon arises when in situ measurements surpass SSM readings at greater soil depths, offering intriguing insights into the underlying influences, which include soil properties, groundwater interactions, and spatial variations. The introduction of a 1-month averaging scheme effectively highlights the potential of this approach to mitigate these discrepancies, resulting in a smoother soil-moisture variation profile and subsequently improving the correlation between SMAP data and in situ measurements.

A significant focus of this study lies in exploring the linear correlation coefficients, characterized by the coefficients M and C. While some stations exhibit unreliable correlations due to factors such as SMAP data volatility and sensor malfunctions, the reliable correlations provide valuable insights into understanding soil-moisture dynamics. The diverse range of calculated correlation coefficients, as indicated by the coefficient of determination (R^2^), underscores the importance of considering the proportionality of measurement areas. Although these correlations are specific to individual stations, their implications extend to site-specific applications and analogous conditions, emphasizing the versatility of remote-sensing data.

Furthermore, the integration of essential soil properties into the correlation analysis through multiple linear regression (MLR) enhances the scientific rigor of this study. This analytical approach illuminates the intricate interconnections between soil characteristics and moisture measurements, revealing the multifaceted nature of soil–water interactions. As a starting point, the resulting regression equations establish simple relationships between linear coefficients (M and C) and basic soil properties (X1, …, X7), thereby shedding light on the underlying mechanisms governing correlations. Nevertheless, it should be noted that the predictive capacity of the MLR method could be limited by the non-linearity and interdependency of soil properties. More sophisticated models, such as machine learning, artificial neural networks, etc., will be explored in our future work.

The imperative of upscaling and correlating soil-moisture data from diverse sources, such as satellite-based SMAP measurements and in situ observations, underscores the need for a comprehensive understanding of soil-moisture dynamics across various scales. The insights gained from this study highlight the pivotal role of measurement area proportions and land-cover types in influencing correlation outcomes. The distinct scales of measurement between SMAP soil-moisture and in situ data, combined with the inherent variations in in situ measurements, impact the correlation results. The role of land-cover type emerges as a significant factor, with croplands displaying enhanced correlations due to their uniformity. Utilizing MODIS land-cover type data allows for the effective classification of telemetry stations, providing insights into the role of land cover in determining correlation efficacy. It should be added that further analyses should focus on the role of vegetation on the correlation development and upscaling model. Additional vegetation indices such as the NDVI will be considered in our future work.

Temporal averaging has emerged as a key technique in bridging the temporal gap between SMAP data and high-frequency in situ measurements. The alignment of the three-month running average with the Oceanic Niño Index (ONI) period resulted in a meaningful agreement between soil-moisture patterns and climate oscillations. Extending this approach to correlate SMAP and in situ soil-moisture data proved effective, particularly when focusing on cropland regions. Through spatial and temporal averaging, robust correlations were achieved, harmonizing diverse data sources into a coherent relationship.

The correlation analysis further uncovered significant insights into the interrelation between SMAP soil moisture (SSM) and in situ water content (θ) at varying depths. By utilizing linear regression models, correlations were established for the average SSM and the in situ θ at depths of 10 cm and 30 cm. The high R-squared values, ranging from 0.83 to 0.87, underline the strong correlations achieved through the three-month running average approach. Notably, distinct patterns emerged from this analysis. At a depth of 10 cm, the SSM consistently exhibits a slight tendency to slightly overpredict in situ moisture (θ), with a systematic transformation shift of around −3%. Importantly, the linear equation’s slope remains close to unity, indicating a proportional correlation. Similarly, at a 30 cm depth, the correlation highlights SSM’s consistent inclination to overpredict the in situ θ, with a slope of the linear equation around 0.53.

While the adjustments we explored did not consistently yield significant improvements in all cases, the positive outcomes observed, particularly for greater soil depths, point toward the potential for refining our models. These results highlight the intricate nature of soil-moisture dynamics in diverse land-cover types, emphasizing the importance of tailored approaches to calibration and adjustment. In our endeavor to create accurate soil-moisture data for climate modeling and weather prediction, this study offers valuable insights and suggests directions for future research. It is clear that the linear adjustment method has promise, but its effectiveness depends on intricate relationships that require further investigation and validation. These endeavors have the potential to deepen our understanding of soil-moisture behavior, contributing to more precise and dependable environmental modeling and predictive systems.

In conclusion, this study signifies a significant advancement in refining soil-moisture estimation by bridging the gap between remote sensing and in situ measurements. The investigation into temporal variations, correlation coefficients, and the role of soil properties culminate in a comprehensive understanding of soil-moisture dynamics. The study underscores the crucial role of temporal averaging and the impact of land-cover types in unraveling the complexities of correlation patterns.

## Figures and Tables

**Table 1:** Location details of telemetry weather/soil-moisture stations used in this study.

No.	Station Name	Province	Latitude	Longitude	Land Use ^1^	Geology ^2^	Geography	Elevation (AMSL)	Average Annual Rainfall ^3^
1	KKCN	Phetchaburi	12.8723	99.6835	Perennial plant	Sedimentary and metamorphic	Plain at the foot of hilly terrains	64 m	983.0 mm
2	VLGE49	Phetchabun	15.61636	101.023	Field crop	Quaternary sediment	Undulating plain	96 m	1210.0 mm
3	HNKA	Chai Nat	14.9696	100.0104	Rice paddy	Quaternary alluvial sediment	Flood plain	13 m	1010.8 mm
4	WGYG	Saraburi	14.8481	101.1459	Field crop	Sedimentary rock	Plain	93 m	1185.2 mm
5	VLGE50	Prachinburi	14.0426	101.7204	Rice paddy	Quaternary alluvial sediment	Alluvial plain	27 m	1762.4 mm
6	NMUB	Nan	18.48421	100.93071	Forest	Sedimentary rock	Undulating plain	333 m	1238.9 mm
7	PAII	Mae Hong Son	19.37012	98.39309	Fruit trees	Granitic	Plain between complex mountain ranges	776 m	1315.8 mm
8	BNKE	Nakhon Phanom	16.90888	104.6187	Rice paddy	Sedimentary and metamorphic	Plain	153 m	2328.0 mm
9	THAT	Surin	15.31667	103.9355	Urban	Sedimentary rock	Plain	128 m	1445.3 mm
10	SWR036	Satun	7.08837	100.002	Perennial plant	Igneous	Basin surrounded by hills	110 m	2386.0 mm

^1^ based on the land use map of the Land Development Department, Thailand; ^2^ based on the geology map of the Department of Mineral Resources, Thailand; ^3^ based on the Thai Meteorological Department.

**Table 2:** Basic soil properties collected from the 10 telemetry stations.

No.	Station Name	Depth (cm)	Atterberg Limits			Grain Size Distribution (%)				Porosity	Organic Content (g/kg)	Specific Gravity	Soil Type (Unified Soil Classification System)
			Liquid Limits (%)	Plastic Limits (%)	Plasticity Index, PI	Gravel	Sand	Silt	Clay				
1	KKCN	0–50	44.70	24.62	20.08	4.24	27.90	21.04	46.83	0.41	20.0	2.64	CL
		50–100	52.80	30.83	21.97	0.71	23.16	26.22	49.91	0.47	5.8	2.41	CH
2	VLGE49	0–100	43.82	25.12	18.70	0.88	37.19	2.03	59.90	0.54	19.0	2.70	CL
3	HNKA	0–10	33.40	18.52	14.88	11.70	66.56	8.29	13.45	0.39	10.0	2.59	SC
		10–40	24.10	14.51	9.59	11.39	43.52	23.29	21.81	0.37	6.8	2.52	SM
		40–100	NP	NP	NP	2.05	63.01	16.87	18.07	0.49	4.9	2.63	SC
4	WGYG	0–50	62.00	48.38	21.63	7.17	40.11	41.49	11.23	0.57	30.0	2.68	MH
		50–100	39.80	29.03	10.77	4.55	44.31	45.44	5.70	0.52	5.8	2.64	ML
5	VLGE50	0–20	25.90	20.16	5.74	2.03	45.73	2.15	50.09	0.45	11.0	2.69	CL
		20–100	29.70	20.79	8.91	3.83	39.61	5.46	51.10	0.38	1.8	2.70	CL
6	NMUB	0–100	NP	NP	NP	17.53	32.04	40.43	10.00	0.58	17.0	2.65	ML
7	PAII	0–10	39.00	28.91	10.09	5.76	43.42	44.32	6.50	0.68	35.0	2.67	ML
		10–100	64.30	26.39	37.91	0.22	43.57	4.20	52.01	0.55	3.5	2.71	CH
8	BNKE	0–50	NP	NP	NP	18.89	46.24	22.26	12.61	0.41	15.0	2.60	SM
		50–70	NP	NP	NP	14.92	50.03	22.13	12.92	0.36	5.8	2.52	SM
		70–100	24.20	16.75	7.45	7.75	48.92	20.80	22.53	0.38	5.3	2.62	SM
9	THAT	0–10	NP	NP	NP	8.19	55.32	15.06	21.44	0.36	10.0	2.60	SC
		10–100	NP	NP	NP	0.07	70.21	13.97	15.74	0.35	0.8	2.49	SM
10	SWR036	0–10	NP	NP	NP	0.25	70.04	16.05	13.66	0.52	15.0	2.55	SM
		10–40	NP	NP	NP	1.46	70.41	12.92	15.22	0.42	8.0	2.48	SC
		40–100	NP	NP	NP	7.51	82.25	4.66	5.57	0.44	1.8	2.60	SP

NP = Non-plastic.

**Table 3:** Moisture-sensor calibration coefficients and hydraulic and thermal properties of the soils.

No.	Station Name	Soil Unit *	Depth (cm)	Sensor Calibration Coefficients			Hydraulic Conductivity, k, cm/s	Thermal Conductivity, λ, W/(m·K)
				a	b	R^2^		
1	KKCN	U	0–50	3.9346	−20.638	0.9839	9.44 × 10^−5^	1.76
		L	50–100	5.5088	−42.002	0.9812	1.32 × 10^−4^	2.34
2	VLGE49	-	0–100	4.9995	−30.271	0.9822	3.31 × 10^−5^	1.68
3	HNKA	U	0–10	3.6222	−22.539	0.9802	1.80 × 10^−7^	1.41
		M	10–40	4.1060	−26.655	0.9872	1.18 × 10^−7^	1.76
		L	40–100	3.6185	−19.873	0.9940	5.83 × 10^−4^	2.01
4	WGYG	U	0–50	4.3506	−26.233	0.9946	2.56 × 10^−4^	1.44
		L	50–100	7.6092	−68.035	0.9847	1.68 × 10^−4^	1.26
5	VLGE50	U	0–20	4.4931	−27.202	0.9911	1.02 × 10^−7^	1.44
		L	20–100	3.5044	−18.562	0.9915	2.61 × 10^−7^	2.01
6	NMUB	-	0–100	6.8022	−56.189	0.9910	2.20 × 10^−3^	1.31
7	PAII	U	0–10	8.2213	−63.759	0.9846	1.33 × 10^−3^	2.63
		L	10–100	6.1297	−38.391	0.9911	2.80 × 10^−6^	1.13
8	BNKE	U	0–50	4.8077	−37.689	0.9947	8.04 × 10^−5^	1.48
		M	50–70	3.5852	−22.114	0.9921	2.56 × 10^−6^	1.67
		L	70–100	4.2354	−29.708	0.9911	4.21 × 10^−7^	2.51
9	THAT	U	0–10	3.5023	−24.270	0.9940	1.62 × 10^−6^	2.87
		L	10–100	3.4499	−22.674	0.9872	1.66 × 10^−5^	2.89
10	SWR036	U	0–10	5.4454	−38.234	0.9944	1.93 × 10^−3^	0.88
		M	10–40	3.8002	−22.982	0.9998	4.32 × 10^−3^	2.34
		L	40–100	5.9551	−39.924	0.9938	4.20 × 10^−3^	1.26

* U = Upper; M = Middle; L = Lower.

**Table 4:** Multiple linear regression coefficients for predicting the sensor calibration equations using soil properties for Equations (2) and (3).

$\alpha_{c}$	$\alpha_{1}$	$\alpha_{2}$	$\alpha_{3}$	$\alpha_{4}$	$\alpha_{5}$	$\alpha_{6}$	$\alpha_{7}$
1598.835	−0.000552	−15.971	−16.009	−15.985	−16.007	14.960	−0.0608
$\beta_{c}$	$\beta_{1}$	$\beta_{2}$	$\beta_{3}$	$\beta_{4}$	$\beta_{5}$	$\beta_{6}$	$\beta_{7}$
−16,579.396	0.123	165.669	166.105	165.639	166.058	−132.139	0.727

**Table 5:** Soil–water retention parameters estimated for the 10 telemetry stations.

No.	Station Name	Soil Unit *	Depth (cm)	Van Genuchten Parameter					
				$\theta_{s}$ (%)	$\theta_{r}$ (%)	p (kPa^−1^)	n	m	R^2^
1	KKCN	U	0–50	41	10	0.072	0.442	1.066	0.958
		L	50–100	47	10	0.496	0.560	1.026	0.960
2	VLGE49	-	0–100	54	20	0.00470	0.318	1.059	0.985
3	HNKA	U	0–10	39	7	0.0524	0.532	1.590	0.948
		M	10–40	37	3	0.00905	0.512	1.350	0.950
		L	40–100	49	2	0.0364	0.413	1.105	0.973
4	WGYG	U	0–50	57	19	0.0211	0.378	1.345	0.969
		L	50–100	52	5	0.0258	0.428	1.207	0.954
5	VLGE50	U	0–20	45	10	0.00216	0.356	1.114	0.962
		L	20–100	38	12	0.0189	0.394	1.168	0.957
6	NMUB	-	0–100	31	2	0.000843	0.484	1.504	0.975
7	PAII	U	0–10	31	15	0.0113	0.906	1.501	0.982
		L	10–100	46	18	0.0370	0.913	0.318	0.936
8	BNKE	U	0–50	30	17	0.00903	0.691	1.758	0.937
		M	50–70	26	6	0.158	0.938	0.309	0.996
		L	70–100	30	9	0.0806	1.075	0.308	0.981
9	THAT	U	0–10	36	6	0.360	4.556	0.0670	0.984
		L	10–100	35	0	0.00311	0.380	1.892	0.971
10	SWR036	U	0–10	17	2	0.164	20.779	0.0181	0.966
		M	10–40	30	0	0.00000133	0.347	17.014	0.989
		L	40–100	25	7	0.115	1.990	0.162	0.967

* U = Upper; M = Middle; L = Lower.

**Table 6:** Linear correlation coefficients for monthly SMAP vs. in situ soil moisture for Stations 1 to 5.

**No.**	1	2		3		4	5		
**Station**	KKCN	VLGE49		HNKA		WGYG	VLGE50		
**Depth (cm)**	all	10	30	10	100	10	10	30	100
**M**	NR	0.3207	0.2883	0.2387	0.3428	0.1283	0.364	0.3451	0.2059
**C**	NR	12.247	42.324	13.493	19.155	42.016	24.438	21.165	29.807
**R^2^**	NR	0.4385	0.3943	0.2086	0.4134	0.1756	0.4971	0.5471	0.5814

NR = Not reliable (negative correlation or R^2^ < 0.1).

**Table 7:** Linear correlation coefficients for monthly SMAP vs. in situ soil moisture for Stations 6 to 10.

**No.**	6			7	8				9		10
**Station**	NMUB			PAII	BNKE				THAT		SWR036
**Depth (cm)**	10	30	60	all	10	30	60	100	30	100	all
**M**	0.4217	1.1006	0.9325	NR	0.4748	0.6607	0.3699	0.3497	0.255	0.3873	NR
**C**	21.306	1.5069	0.4633	NR	9.0514	2.7021	12.625	21.448	5.6962	4.0059	NR
**R^2^**	0.1296	0.5333	0.5378	NR	0.5247	0.6376	0.4906	0.4722	0.2969	0.3345	NR

NR = Not reliable (negative correlation or R^2^ < 0.1).

**Table 8:** Multiple linear regression coefficients for predicting the sensor calibration equations using soil properties for Equations (6) and (7).

D	$a_{1}$	$a_{2}$	$a_{3}$	$a_{4}$	$a_{5}$	$a_{6}$	$a_{7}$
−400.298	−0.00987	4.0132	4.0052	4.0042	4.0083	0.1167	0.0006060
E	$b_{1}$	$b_{2}$	$b_{3}$	$b_{4}$	$b_{5}$	$b_{6}$	$b_{7}$
−219,718.912	0.083	2196.216	2197.531	2198.317	2197.613	−54.224	0.377

## Data Availability

Readers can find the available data on the SMAP soil moisture at https://smap.jpl.nasa.gov/ (last accessed: 13 June 2023). The in situ soil-moisture data from Thailand can be found by contacting the corresponding author.

## List of Notations

SMAP	Soil Moisture Active/Passive
**θ**	In situ volumetric water content
SSM	Surface soil moisture
$V_{w}$	Volume of soil water
V	Total volume of soil
x	Sensor reading (mA)
a, b	Fitting parameters for water content–voltage sensor calibration from linear regression
$\alpha_{c}$, $\alpha_{1},.,\alpha_{7}$	Multiple linear regression parameters for sensor calibration based on physical properties for a
$\beta_{c}$, $\beta_{1},.,\beta_{7}$	Multiple linear regressions parameters for sensor calibration based on physical properties for b
$X_{1},.,X_{7}$	Soil physical properties used in multiple linear regressions
$\theta_{s}$	Saturated volumetric water content
$\theta_{r}$	Residual volumetric water content
s	Soil suction
p, m, n	Van Genuchten’s fitting parameter for soil–water retention curves
M, C	Linear regression fitting parameters between θ and SSM, based on 1-month average values
D, $a_{1}$, …,$a_{7}$	Multiple linear regression parameters for M, based on physical properties
$E,b_{1},\ldots ,b_{7}$	Multiple linear regression parameters for C, based on physical properties
